# Recent Advances and Novel Approaches in Laboratory-Based Diagnostic Mycology

**DOI:** 10.3390/jof7010041

**Published:** 2021-01-11

**Authors:** Lewis P. White, Jessica S. Price

**Affiliations:** Mycology Reference Laboratory, Public Health Wales, Microbiology Cardiff, Cardiff CF14 4XW, UK; Jessica.Price2@wales.nhs.uk

**Keywords:** mycology, novel targets and applications, next-generation sequencing, advances in serology, advances in molecular diagnostics

## Abstract

What was once just culture and microscopy the field of diagnostic mycology has significantly advanced in recent years and continues to incorporate novel assays and strategies to meet the changes in clinical demand. The emergence of widespread resistance to antifungal therapy has led to the development of a range of molecular tests that target mutations associated with phenotypic resistance, to complement classical susceptibility testing and initial applications of next-generation sequencing are being described. Lateral flow assays provide rapid results, with simplicity allowing the test to be performed outside specialist centres, potentially as point-of-care tests. Mycology has responded positively to an ever-diversifying patient population by rapidly identifying risk and developing diagnostic strategies to improve patient management. Nowadays, the diagnostic repertoire of the mycology laboratory employs classical, molecular and serological tests and should be keen to embrace diagnostic advancements that can improve diagnosis in this notoriously difficult field.

## 1. Introduction

The impact of fungal disease is greatly overlooked, but with an ever-increasing immunosuppressed or immune-modulated (monoclonal antibody therapies) at-risk population, coupled with inevitable exposure to fungi and the availability of more sensitive diagnostics, cases of fungal disease will inevitably increase [1]. While the world struggles to cope with the COVID-19 pandemic, not unexpectedly, fungi have exploited the situation, causing severe, secondary invasive fungal disease (IFD) in critically ill COVID-19 patients, highlighting the difficulty of diagnosing IFD in the heterogeneous intensive care unit (ICU) population [2,3,4]. Given the now well documented association between invasive aspergillosis (IA) post severe influenza infection, the critically ill patient with severe respiratory virus infection represents a significant patient population that previously would not have been considered at risk of IA. Indeed, the incidence of IFD in the ICU may be significantly overlooked and while *Candida* is generally accepted as the most likely cause of critical-care IFD, the significance of IA (incidence: 12.4%) was recently highlighted in a multicentre, UK-based study of 194 mechanically ventilated ICU patients, lengthening ICU stay by a median of 10 days and potentially increasing mortality [5]. While nonculture biomarker diagnostics have been extensively validated in specific populations (e.g., haematology), their performance in the heterogeneous ICU population requires further validation before a true incidence of IFD in the ICU is determined and the clinical benefits of novel testing can be utilized.

The field of mycology has seen many developments in recent years. Fungal PCR tests have been extensively standardized, complemented by the release of commercial assays allowing such tests to be performed in non-specialist centres. Antifungal resistance in commonly diagnosed genera (e.g., *Candida* and *Aspergillus* spp.), is clinically concerning and assays to rapidly identify this, while overcoming classical limitations, have been developed. Next-generation sequencing is providing an insight into the mycobiome, fungal evolution, host-fungi interactions, identifying novel and existing resistance mechanisms and potential antifungal targets, and may have a role in screening the host to identify a genetic predisposition to IFD and provide personal medicine that pre-empts infection. 

This review will summarize recent diagnostic developments and application of novel methods in diagnostic mycology.

## 2. Advances in Molecular Diagnostics

Standardization of methods, the availability of commercial PCR tests and external quality assurance schemes (Quality control for Molecular diagnostics (QCMD)) have significantly improved the acceptance and broadened the use of molecular methods to aid the diagnosis of aspergillosis, candidiasis, mucormycosis and *Pneumocystis* pneumonia (PcP) [6,7,8,9,10,11,12]. What was once a test to be performed by specialist mycological laboratories, methodological simplicity and commercial tests provide processes that are comparable to molecular assays for the detection of virological pathogens, permitting use in general molecular diagnostic facilities.

The development of fully automated, commercial molecular platforms capable of directly processing vacutainers for the presence of fungi, further widens the opportunity for enhancing fungal diagnostics, although the high testing costs of platforms, such as the T2Candida, may limit widespread application, irrespective of performance. While it appears that the T2Candida will provide sufficient sensitivity to exclude invasive candidosis (IC) when negative (NPV: ≥98), data are conflicting, with the MADRID study demonstrating limited sensitivity and NPV (36%/80%), albeit during empirical AFT and further real-life clinical data are required [13,14,15]. Studies indicate the T2Candida is useful for predicting patient prognosis through source control, allowing de-escalation of treatment and determining treatment duration [14,15,16]. The availability of alternative commercial *Candida* PCR assays, although not automated, may be incorporated into current molecular diagnostic facilities with greater ease over the T2Candida and while initial performance of assays is good (Bruker Fungiplex Candida Se: 100%, Sp: 94%), further evaluation is required [17]. The fully automated detection of PcP has been performed using the BD MAX™ system, where a prospective evaluation of BAL fluid testing generated sensitivity/specificity of 93%/95% and provided Cq values that could differentiate colonization from infection [18]. It is unclear whether this system will provide sufficient analytical sensitivity for the detection of other IFD, given the small PCR reaction volume limiting DNA template input. Microfluidic PCR platforms have shown limited sensitivity (56%) for the detection of culture proven candidaemia [19].

### 2.1. Molecular Detection of Antifungal Resistance

The emergence of IFD resistant to antifungal therapy is clinically very concerning, given the limited number of antifungal classes. Molecular techniques remain the only nonculture method capable of identifying potential resistance through screening for genetic markers (single nucleotide polymorphisms (SNPs) or tandem repeat sequences) or fungal species associated with inherent resistance. These molecular approaches will possibly enhance sensitivity and turn-around time, albeit without providing MIC data. PCR assays have been developed for the detection of *C. auris*, to the permit the fully automated processing of skin and environmental swabs using the T2 platform [20]. Other commercial assays have been used for processes outside the original design scope, with the Pathonostics Aspergenius real-time PCR test being adapted to differentiate potentially resistant species within the *A. fumigati* complex [21].

Initially, the identification of mutations associated with antifungal resistance was identified by various “in-house” molecular methods and/or sequencing of target genes (e.g., *cyp*51A in *A. fumigatus*) [22]. More recently real-time PCR assays have been employed, but are generally only designed to detect the major causes of resistance within individual species. However, they are capable of detecting multiple different mutations associated with resistance and identifying resistant strains in heterogeneous populations predominantly composed of sensitive strains [23]. A concept which will be clinically useful when testing respiratory samples from patients with chronic conditions (e.g., Cystic Fibrosis). Nowadays, commercial assays are available to detect the most frequent markers of resistance in commonly encountered fungal pathogens. Assays, such as the Pathonostics Aspergenius^®^ is designed to detect the most frequent mutations conferring azole resistance (TR34/L98H, TR46/Y121F and TR46/T289A) in *A. fumigatus* and has been applied to direct specimen testing [22,24,25]. Similarly, detection of resistance in *Pneumocystis jirovecii* is feasible through the Pathonostics PneumoGenius^®^, which identifies dihydropteroate synthase (DHPS) mutations responsible for resistance to sulfa-based drugs (sulfamethoxazole and dapsone) [26]. The use of loop-mediated isothermal amplification (LAMP) is better suited for the molecular detection of antifungal resistance in resource limited settings, with the added potential of conversion into point of care devices. A TR34 LAMP assay has been developed for the detection of azole resistance in *A. fumigatus*, generating rapid results, with the colorimetric change interpreted by the naked eye or using a smart phone with image analysis software and showed good discrimination when testing clinical isolates [27].

Pyrosequencing is an alternative method, with the potential to screen for all known mutations within specific target genes and has been successfully applied to the testing of respiratory samples from patients with chronic pulmonary aspergillosis [28,29]. A surveyor nuclease assay has also been designed to detect point mutations associated with azole resistance in the *cyp51A* gene of *A. fumgiatus* [30]. Utilizing surveyor nuclease to cleave heteroduplex DNA, specifically at base mismatch sites, the approach has the potential to detect a wide range of different point mutations, albeit taking significantly longer than real-time PCR detection.

While these approaches enhance routine diagnostic capabilities, it is critical that conventional susceptibility testing continues and is combined with molecular surveillance for novel mechanisms of resistance and genetic drift, likely through next-generation sequencing (NGS), performed in reference facilities.

### 2.2. Droplet Digital PCR

Droplet digital PCR (DD-PCR) is a novel technique that segregates the PCR reaction mix a thousand-fold, allowing PCR amplification and fluorescent end-point quantification in each individual droplet [31]. Poisson statistics are applied to the fraction of positive and negative droplets to estimate the total number of copies in the input sample without the need for a standard curve. The method can be multiplexed, is tolerant to inhibition, has a lower detection limit, is highly reproducible and may prove beneficial for the detection of pathogens present at the limit of standard real-time PCR detection (e.g., fungal DNA in blood), where it can provide absolute quantification and screening for mutations (e.g., antifungal resistance). The main drawback of DD-PCR is poor performance when testing samples with high (>10^5^) copy numbers, where the accuracy of copy number calculations can be affected and sample dilution may be necessary [32].

DD-PCR has been used to detect *A. fumigatus* and *A. terreus* in respiratory specimens, where a duplex PCR targeting the ITS region generated improved sensitivity and lower rates of inhibition compared to similarly designed real-time PCR [32]. DD-PCR targeting the 28S rRNA of *A. fumigatus* generated higher copy numbers compared to a real-time PCR when testing DNA from isolates, but did not improve sensitivity when testing serum samples from cases of IA [33]. The concept of DD-PCR represents an exciting development in molecular diagnosis, as its potential for detection of lower copy numbers could be particularly beneficial to fungal PCR testing of blood samples. At this time, it is not possible to confirm its suitability and further evaluations are required.

### 2.3. Next-Generation Sequencing

With fungi likely critical for both a balanced microbiome and typical commensal interactivity with the host, NGS could play a crucial role in identifying compositional changes in the microbiome/mycobiome or genetic predisposition in the host, which may lead to enhanced understanding of disease pathology while providing personalized medicine. Additionally, the sequencing of whole genomes from DNA isolated from individual isolates can be used to advance phylogenetic studies, identify existing and novel genetic markers of antifungal resistance or conversely novel targets for antifungal therapy and provide epidemiological evidence of transmission when investigating potential clinical outbreaks.

To date the mycobiome of the oral cavity, skin, gastrointestinal, respiratory and genital tract have been investigated using NGS [34]. NGS studies of the human mycobiome show wide diversity (>390 fungal species) that varies according to the anatomical location and subsequent differing environmental conditions (e.g., respiratory versus gastrointestinal tract), but most studies confirm the significant limitations of culture based evaluations of the mycobiome, leading to significantly reduced biodiversity and failure to detect the entire microbiological population; limiting our understanding on the interactions between the host and microbiome [35,36]. In patients with underlying conditions associated with increased risk of fungal infection (e.g., HIV and Cystic Fibrosis) NGS has shown increased levels of potentially pathogenic fungi, such as *Aspergillus*, in the mycobiome of the respiratory tract and oral cavity [37,38]. During IFD, a decrease in mycological diversity in the infected anatomical areas has been documented [39].

Investigations of the mycobiome using molecular methods are fallible. While the direct application of whole genome sequencing of biological specimens, such as BAL fluid, is preferable, the microbiological complexity and the limited abundance (<1%) of fungal species in the microbiome requires the incorporation of PCR amplification of specific genes (usually rRNA gene cluster) to provide sufficient sequencing depth [36]. However, amplification of these multicopy targets (e.g., internal transcribed spacer (ITS) regions) may not provide sufficient resolution for species level identification of fungi, and while pan-fungal amplification is ideal for mycobiome studies, it can limit clinical applications and complicate interpretation [40].

For instance, *Candida* species were identified by NGS as the dominant fungi in the respiratory tract, a fact already determined by culture [35]. As *Candida* are commensals of the respiratory tract their clinical significance is usually limited, despite their relative abundance. The performance of NGS utilizing pan-fungal PCR amplification of ribosomal RNA genes may be affected by the overwhelming presence of a single species, utilizing excessive amounts of molecular reagents during amplification and limiting the detection of fungi that are clinically important but potentially present in lesser burdens. Pan-fungal detection is required for mycobiome studies, but the application of NGS in the clinical setting needs to be designed to overcome the detection of probably insignificant commensal fungi while targeting more likely pathogens.

The practical limitations of NGS for the study of the mycobiome have been thoroughly discussed by Nilsson et al. [41]. As with any molecular-based investigation, they emphasize the prerequisite for optimal sample preparation, including controlling for procedural fungal contamination, appropriate sample storage prior to testing to limit fungal overgrowth and utilizing optimal nucleic acid extraction protocols. Combining this with efficient PCR amplification (carefully selected primers and a high-fidelity PCR polymerase) is critical, and the utilization of positive and negative controls and understanding the biases introduced throughout the process is essential when interpreting NGS data [41].

NGS has been used to investigate the mechanisms of resistance to antifungals [42,43]. The *cyp51A* gene in *A. fumigatus* has been screened using high-resolution analysis to identify SNPs associated with azole resistance, confirming the TR_34_/L98H mutation as the most common SNP associated with azole resistance [42]. In *Candida* species, NGS has also been used to investigate six genes (ERG11, ERG3, TAC1, CgPDR1, FKS1 and FKS2) commonly associated with antifungal resistance to azoles and echinocandins [43]. Ninety-one SNPs, six being novel, were identified [43]. Evolutionary studies have utilized NGS to demonstrate significant genetic diversity in clinical *A. fumigatus* resistant isolates within certain countries, although this is not consistent and in India data indicated a selective sweep of a single, significantly related, highly fit resistant genotype [42].

With a significant number of different host mutations associated with increased risk of developing IFD, NGS genetic screening of susceptible patients for mutations predisposing them to a specific fungal disease is possible [44,45]. Given the range of mutations and the fact that most patients will be susceptible to a range of infections, including non-fungal infections, it makes sense to utilize whole genome sequencing to identify a broad range of enhanced genetic risk. While predictive modelling mentioned using real-time PCR for screening for five SNPs associated with IA represents significant progress in personalized medicine, host mutations associated with aspergillosis exceeds what can be conveniently screened using non-sequencing-based methods, with sequencing, including NGS needed to identify novel mutations [44,46]. While microarrays and genome wide SNP analysis have identified genetic risk factors, NGS of the human genome will provide an extensive clinical information beyond mycology and is a method that will enhance clinical practice and personalized medicine.

## 3. Advances in Serological Diagnosis

### 3.1. New Approaches for the Detection of (1-3)-β-D-Glucan

One of the major hurdles to the widespread use of plate based (1-3)-β-d-Glucan (BDG) was the necessity to test larger numbers of samples to minimize wastage that drove up testing costs. Recently, the Associates of Cape Cod (responsible for the Fungitell BDG assay) developed the Fungitell STAT™ assay that permits simple, rapid (<1 h) testing of 1–7 samples per run [47]. The assay removes the need to run multiple standards, and uses a single calibrator to provide qualitative index values, representative of positive, indeterminate and negative results. Comparison with the original Fungitell generated good correlation between index and BDG concentration when testing patient (correlation coefficient: 0.90) and contrived specimens (correlation coefficient 0.99). While overall positive and negative agreement between the original Fungitell and STAT was excellent (>95%), not unexpectedly, most discordance occurred for results at the thresholds of qualitative interpretation [47]. Importantly between run and between laboratory STAT reproducibility was comparable to the original Funigtell. Further multicentre clinical performance validation is required.

An alternative approach to BDG testing of limited sample numbers can be provided by the Fuijfim Wako B-Glucan test. Recent evaluations comparing the Wako and Fungitell assays to aid in the diagnosis of *Pneumocystis* pneumonia (PCP) and IC, indicated that while the Wako assay provided better specificity, it lacked the sensitivity of the Fungitell assay [48,49]. In both studies reducing the positivity threshold of the Wako assay improved sensitivity while compromising specificity. This concept was taken further by De Carolis et al. who confirmed the need to use a lower positivity threshold when using the Wako assay to test 40 cases of IA, 78 cases of IC and 17 cases of PCP, lowering the threshold improved sensitivity between 6–20% across infections, while specificity remained excellent (97.3%, *n* = 187 controls) [50].

The availability of simple to perform BDG assays, suitable for the testing of lesser numbers may permit more centres to offer this test, improving accessibility and turn-around time.

### 3.2. Developments in Enzyme Immunosorbent Assays for the Detection Galactomannan

Despite galactomannan enzyme immunoassay (GM-EIA) testing being performed using a single commercial assay (Biorad *Aspergillus* Ag kit) for many years, performance has been highly variable between centres [51]. It is not clear from where this heterogeneity arises, given the methodology is consistent, but it could reflect the influence of manual testing. The ability to automate GM-EIA testing using the BioRad Evolis instrument limits the impact of the human variable, permits screening of large numbers and allow two different assays (e.g., GM-EIA and *Aspergillus* IgG) to be run concurrently, although published data specific to the GM-EIA are not currently available. However, it is not necessary to purchase bespoke instrumentation, and more generic platforms (e.g., Dynex DS2 ELISA processing system) have been programmed to perform the GM-EIA assay [52]. In a multicentre study, the use of the DS2 reduced variability when compared to manual GM-EIA, the detection of positive samples from cases of IA were unaffected while there was a significant decrease in GMI for control-based samples, leading to a reduction in potential false positive results [52].

Recently, a novel GM-EIA assay developed by IMMY generated a sensitivity and specificity of 71% and 98%, respectively, when testing serum using the conventional positivity threshold of 0.5 [53]. Currently, no optimal positivity threshold has been defined for this assay, nevertheless its performance using a 0.5 threshold is comparable to the widely used BioRad GM-EIA. Reducing the threshold to ≥0.27 generated sensitivity and specificity of 90% and 92%, respectively. Agreement between the IMMY and BioRad GM-EIA was excellent (observed agreement 95%, Kappa: 0.82), and the IMMY GM-EIA appears to be a comparable alternative to the Bio-Rad GM-EIA when testing serum samples [53]. An alternative ELISA assay targeting a novel *Aspergillus* specific galactomannoprotein was also compared to the well-established BioRad GM-EIA [54]. Overall, sensitivity for both assays when testing serum was poor (40–56%), but likely reflected the sampling strategy employed with serial screening lacking, while specificity was excellent >95%. Concordance between the two assays was good (observed agreement 86%, Kappa: 0.64). The development of alternative GM-EIA tests by different manufacturers could, as a result of market forces, lead to cost savings for service users and pressurize manufacturers to improve product design, procedure and hopefully, performance.

### 3.3. Lateral Flow Devices for the Diagnosis of IFD

Lateral flow devices (LFD) to detect fungal antigens are not novel. The cryptococcal LFD has revolutionized the diagnosis of crytpococcosis, particularly in resource limited settings where a simple assay, requiring rudimentary laboratory facilities and limited training has provided excellent sensitivity (>98%) and specificity (>98%) when testing serum or cerebral spinal fluid [55].

The first LFD for IA was described in 2008, meta-analytical performance, primarily focussed on a single, pre-CE marked version of the OLM Diagnostics device, generated pooled sensitivity and specificity of 68% and 87%, respectively, when testing serum, and 86% and 93%, respectively, when testing BAL fluid [56,57]. The release of the IMMY Sona *Aspergillus* Galactomannan lateral flow assay (LFA) represents a potentially exciting development in the field of mycology. The design concept of incorporating two monoclonal antibodies (Mab), one novel Mab and one targeting a similar GM epitope to the BioRad *Aspergillus* antigen assay, has the potential to improve performance as demonstrated with the cryptococcal LFD. Studies focusing on the performance of the LFA when testing respiratory samples (e.g., BAL fluid), generated sensitivity and specificity of 83–92% and 91–92%, respectively [58,59]. Comparison with the OLM LFD when testing BAL fluid showed the LFA to provide significantly better sensitivity (83% versus 69%, *p* = 0.008), while maintain specificity (87%) for the diagnosis of proven/probable IA (*n* = 75) [58]. As with other biomarker tests the use of antifungal therapy was shown to affect performance, significantly reducing sensitivity (88% vs. 63%, *p* = 0.009). The retrospective performance of the LFA when testing serum was compared to the BioRad GM-EIA [60] LFA sensitivity/specificity, utilizing the typical positivity index of 0.5, was 97%/98%, respectively, and the LFA was positive in four cases of probable IFD (radiology typical of IA, BDG positive but GM-EIA negative) that appear to have been missed by the BioRad GM-EIA. Across cases and controls, the galactomannan index values generated by the LFA were greater than the GM-EIA, indicating that the approach of combining two Mab enhanced signal strength, but was not necessarily associated with significant false positivity [60]. The application of digital readers—which provide a quantitative output are a major improvement when using LFD, removing subjectivity and providing a value for clinical interpretation and have been identified as critical to clinical use, can be applied to both serum and BAL fluid, and are available for both the IMMY and OLM assays.

The detection of urine antigens specific to *Aspergillus* using a lateral flow assay optimized with the galactofuranose specific monoclonal antibody (mAb476) generated sensitivity and specificity of 80% and 92%, respectively, and provides an alternative easily obtained sample for the screening of IA [61]. Further clinical evaluation, including testing of more typical sample types is required.

### 3.4. Proximity Ligation Assay for Invasive Aspergillosis

Proximity ligation assays (PLA) combine the sensitivity of a molecular testing with the specificity of antibody detection. A PLA utilizing two biotinylated proximity probes (A+B) based on the *Aspergillus* specific monoclonal antibody JF5 (used in the OLM *Aspergillus* LFD) targeting antigenic mannoproteins has been designed [62]. The probes link to two different, non-complementary streptavidin labelled DNA oligonucleotides, when they bind to adjacent epitopes the oligonucleotides form a single DNA strand that can be PCR amplified and detected using qPCR. Currently, most validation is analytical, limited to the testing *Aspergillus* culture filtrate or contrived samples, but the limit of detection of the PLA was superior to that of the LFD (100-fold) and GM-EIA (10-fold) when testing serum. Clinical evaluation generated sensitivity and specificity of 100%, but numbers were small (4 cases and 10 controls) and currently limited to the testing of BAL fluid [62] Further, extensive validation is required to determine if the potential of this method can be met.

## 4. Novel Targets

### 4.1. Novel Targets for Mucormycosis

Despite improvements in the standardization of PCR for the diagnosis of Mucorales species, the diagnosis of mucormycosis relies heavily on classical mycology. The absence of antigenic markers, whether specific or pan-fungal and inclusive of mucorales species, is a significant hurdle to improving the diagnosis of this devastating infection. The discovery of a pan-fungal dihexasaccharide, inclusive of Mucorales species is significant, particularly as disaccharide detection utilizes matrix-assisted laser desorption ionization—time of flight (MALDI-TOF) mass spectrometry (MS) [63]. The sensitivity for the diagnosis of mucormycosis was 90%.

A further development to improve the diagnosis of mucormycosis was the identification of *Rhizopus oryzae* specific 23 kDa protein using signal sequence trap retrovirus mediated expression (SST-REX) [64]. The presence of the antigen was determined in serum and lung homogenates from an animal model, using an ELISA method. Antigen levels were significantly greater in serum from infected mice (161.1 ng/mL) compared to the controls (57.7 ng/mL) and in lung homogenates (Infected mice: 307 ng/mL; Controls 66.1 ng/mL) [64]. Given mucormycosis can be caused by a wide range of genera/species, the confirmation of a broader detection range is paramount.

A recent evaluation for the detection of this antigen in human serum found levels were higher in cases of mucormycosis (15.1 ng/mL) compared to cases of IA (0.53 ng/mL) and controls without IFD (0.49 ng/mL) [65]. For both these methods, further evaluations to determine the robustness and clinical performance are required.

### 4.2. Novel Targets for Aspergillosis

The secretion of proteases in biological specimens has been investigated as a potential novel diagnostic strategy [66]. Using an animal model, internally quenched fluorogenic probes were utilized to detect proteolytic activity associated with infection, and comparative profiling identified the reproducible activity of prolyl endopeptidase in infected animals. Fluorescent probes specific to the prolyl endopeptidase substrate motif were employed and were capable of detecting infection in animals that was comparable to existing diagnostic tests, but varied between *A. fumigatus* strains [66]. The identity, range of cross *Aspergillus* species expression and direct association with human infection of any endopeptidase to be used in diagnostic capacity must be confirmed before any assay can be developed, and so considerable further development remains. The pan-fungal dihexasaccharide described above has also been evaluated for the diagnosis of IA, generating sensitivity and specificity of 93% and 76%, respectively, with MALDI-TOF MS positivity prior to GM-EIA in 40% (6/15) of patients [63].

With most diagnostic fungal PCR methods targeting the multi-copy rRNA gene cluster to enhance analytical sensitivity, the use of other genes is often limited to improve species differentiation or for the detection of specific mutations (e.g., associated with antifungal resistance). The aspHS gene encodes a haemolysin that is overexpressed during infection and can be used to differentiate between germinated and non-germinated conidia, providing the potential to overcome one of the major limitations when performing *Aspergillus* PCR testing of respiratory samples [67]. Unfortunately, the analytical specificity of a real-time PCR targeting this gene was restricted to the detection of *A. fumigatus*, and while this remains the most common cause of IA, the ability to detect other *Aspergillus* species is critical [68]. When testing BAL fluid from 12 cases of proven/probable IA significant inhibition (42%), likely associated with an inefficient DNA extraction protocol undermined the interpretation of performance, although positivity in tissue biopsies was associated with the presence of hyphal invasion [67].

## 5. Novel Approaches

### 5.1. Assays Targeting fungal Specific T-Cells

The detection of fungal specific functional T-cells by flow cytometry, ELISPOT or ELISA have been proposed as a potential target for the rapid identification of IFD [69]. Initial studies on *Aspergillus* and Mucorales specific T-cells have provided high sensitivities and specificities, and an indication of prognosis [69,70]. Unfortunately, pre-test conditions such as storage, cryopreservation and thawing can influence T-cell assay performance, and require standardization and optimization for these tests to performed routinely, particularly given it is likely that testing will only be performed in specialist centres [71]. Research is on-going to deliver this detail and provide the robust testing required for routine service.

### 5.2. Detection of Galactomannan in Exhaled Breath Condensate

The detection of volatile organic compounds (VOC), such as individual 2-Pentylfuran or a specific pattern of sensor signals (breathprint), in exhaled air using electronic nose (eNose) technology is not novel but represents non-intrusive respiratory test for the diagnosis of IA [72,73]. Unfortunately, clinical validation is limited and has not progressed significantly, possibly a result of the difficulty in replicating individual breathprints. The detection of a well-established *Aspergillus* biomarker, such as galactomannan, using already existing methods (GM-EIA) in exhaled breath condensate (EBC) could be accepted more widely, should performance be satisfactory. A recent prospective evaluation comparing the performance of the GM-EIA testing over 400 BAL fluids and EBC from lung transplant recipients and haematology patients showed that while EBC GMI values from patients were significantly greater than those from health volunteers, no association between IA and a raised EBC GMI was determined, ROC analysis showed no apparent diagnostic benefit [74]. While it appears that galactomannan is detectable in EBC, correlation to a clinical diagnosis of IA may be impeded by our limited understanding of the properties of EBC and optimal sampling processes specific to galactomannan.

### 5.3. MALDI-TOF for Identifying Antifungal Resistance

While susceptibility testing using conventional micro-dilution methods is generally accurate, it adds further delay post recovery of a positive fungal culture. Rapid detection of antifungal resistance using MALDI-TOF MS in cultured yeasts associated with invasive disease may enhance clinical utility of susceptibility testing through the earlier administration of appropriate therapy in resistance cases [75]. Initial studies compared MALDI-TOF MS spectra in yeasts after exposure to various antifungal concentrations to generate a composite correlation index used to determine resistance associated with growth in the presence of intermediate/high antifungal concentrations. Agreement with conventional micro-dilution varied, dependent on drug and species tested, and the time saving associated with the MALDI-TOF MS method was modest compared to conventional methods [75]. 

Resistance to caspofungin in *C. albicans* and *C. glabrata* has been documented using the technique MALDI Biotyper antibiotic susceptibility test rapid assay (MBT ASTRA), where growth in the absence or presence of caspofungin is monitored by MALDI-TOF MS, categorical agreement with CLSI microdilution was 100% for *C. albicans* when growth in the control wells was sufficient, while for *C. glabrata* MBT ASTRA generated higher rates of resistance [76].While the requirement for culture in the presence of various concentrations still incurs a time delay, the time associated with MBT ASTRA was significantly less than conventional microdilution methods (6 versus 24–48 h).

### 5.4. (1-3)-β-d-Glucan as a Prognostic Marker

During IFD, the presence of BDG is recognized by various immune cells, primarily through dectin-1 receptors, which instil a pro-inflammatory (Th-1/Th-17) response. BDG false positivity may arise from a number of clinical and non-clinical sources, including translocation from the gastrointestinal tract and while clinically not associated with IFD, can still lead to an inflammatory response, resulting in worsening sepsis and neurocognitive impairment [77,78]. The presence of BDG (>100 pg/mL) post abdominal surgery, was associated with an increased sequential organ failure assessment (SOFA) score and increased mortality (14% versus 39%), particularly if the BDG concentration persisted >100 pg/mL (mortality 48%) [79]. While BDG positivity was indicative of IC, its absence (<100 pg/mL) despite documented IC was associated with lower mortality (25%) compared to a higher BDG concentration (>100 pg/mL) in the absence of IC (37%). Clinically, this represents a particularly difficult scenario to manage, while IFD can be controlled through antifungal therapy, it is not always feasible to identify and control a non-infective BDG source. It also has implications in clinical trials, where the presence of non-infective BDG and subsequent pro-inflammatory response may have a deleterious effect on patients and potentially undermine studies.

Conversely, BDG negativity is less likely to be associated with IFD and severe clinical presentations leading to lower 30-day mortality [80]. Persistent BDG negativity was independently associated with better prognoses in multivariate analysis (odds ratio 0.12, 95% CI: 0.03–0.49) and has been shown as a safe strategy to base antifungal stewardship schemes [80,81].

## 6. Conclusions

The methodological standardization, availability of commercial Fungal PCR assays and external quality control schemes have permitted the inclusion of molecular tests into the second revision of the EORTC/MSGGERC definitions for classifying IFD and will likely enhance the use of fungal PCR testing making it comparable with antigen testing. [82] The broader range of PCR applications over antigen testing will further enhance their appeal and the availability of commercial molecular tests capable of detecting resistant species (e.g., *Candida auris*) or genetic markers associated with resistance (e.g., TR34/L98H in *A. fumigatus*) may prove beneficial when managing subsequent cases, by overcoming the limitations of conventional mycology and antigen testing. While NGS offers many potential benefits to the mycological field, it is critical that efficient direct sample testing is feasible, otherwise any benefits will be restricted and unduly influenced by the current limitations, but NGS should also be focused on the host to identify genetic predispositions to IFD. The availability of rapid lateral flow tests, with the potential for point of care testing, and development of more cost-effective testing strategies will allow specific diagnostics to move outside the specialist mycology setting, which is important given the ever-increasing population of patients at risk of IFD, as demonstrated in the on-going COVID-19 pandemic. While novel fungal targets have been identified, the novel application of existing methods to test new specimen types, provide alternative information regarding the infection or clinical prognosis are equally important when moving fungal diagnostics forward.

## References

[1] Nedel W.L., Kontoyiannis D.P., Pasqualotto A.C. (2009). Aspergillosis in patients treated with monoclonal antibodies. Rev. Iberoam. Micol..

[2] White L., Dhillon R., Cordey A., Hughes H., Faggian F., Soni S., Pandey M., Whitaker H., May A., Morgan M. (2020). A National Strategy to Diagnose COVID-19 Associated Invasive Fungal Disease in the ICU. Clin. Infect. Dis..

[3] Arastehfar A., Carvalho A., Nguyen M.H., Hedayati M.T., Netea M.G., Perlin D.S., Hoenigl M. (2020). COVID-19-Associated Candidiasis (CAC): An Underestimated Complication in the Absence of Immunological Predispositions?. J. Fungi.

[4] Armstrong-James D., Youngs J., Bicanic T., Abdolrasouli A., Denning D.W., Johnson E., Mehra V., Pagliuca T., Patel B., Rhodes J. (2020). Confronting and mitigating the risk of COVID-19 associated pulmonary aspergillosis. Eur. Respir. J..

[5] Loughlin L., Hellyer T.P., White P.L., McAuley D.F., Conway Morris A., Posso R.B., Richardson M.D., Denning D.W., Simpson A.J., McMullan R. (2020). Pulmonary Aspergillosis in Patients with Suspected Ventilator-associated Pneumonia in UK ICUs. Am. J. Respir. Crit. Care Med..

[6] White P.L., Bretagne S., Klingspor L., Melchers W.J.G., McCulloch E., Schulz B., Finnstrom N., Mengoli C., Barnes R.A., Donnelly J.P. (2010). Aspergillus PCR: One Step Closer to Standardization. J. Clin. Microbiol..

[7] White P.L., Mengoli C., Bretagne S., Cuenca-Estrella M., Finnstrom N., Klingspor L., Melchers W.J.G., McCulloch E., Barnes R.A., Donnelly J.P. (2011). Evaluation of Aspergillus PCR Protocols for Testing Serum Specimens. J. Clin. Microbiol..

[8] Loeffler J., Mengoli C., Springer J., Bretagne S., Estrella M.C., Klingspor L., Lagrou K., Melchers W., Morton C.O., Barnes R.A. (2015). Analytical Comparison ofIn Vitro-Spiked Human Serum and Plasma for PCR-Based Detection of Aspergillus fumigatus DNA: A Study by the European Aspergillus PCR Initiative. J. Clin. Microbiol..

[9] White P.L., Barnes R.A., Springer J., Klingspor L., Cuenca-Estrella M., Morton C.O., Lagrou K., Bretagne S., Melchers W.J.G., Mengoli C. (2015). Clinical Performance of Aspergillus PCR for Testing Serum and Plasma: A Study by the European Aspergillus PCR Initiative. J. Clin. Microbiol..

[10] White P.L., Wingard J.R., Bretagne S., Löffler J., Patterson T.F., Slavin M.A., Barnes R.A., Pappas P.G., Donnelly J.P. (2015). Aspergillus Polymerase Chain Reaction: Systematic Review of Evidence for Clinical Use in Comparison with Antigen Testing. Clin. Infect. Dis..

[11] Rocchi S., Scherer E., Mengoli C., Alanio A., Botterel F., Bougnoux M.E., Bretagne S., Cogliati M., Cornu M., Dalle F. (2020). Interlaboratory evaluation of Mucorales PCR assays for testing serum specimens: A study by the fungal PCR Initiative and the Modimucor study group. Med. Mycol..

[12] Gits-Muselli M., White P.L., Mengoli C., Chen S., Crowley B., Dingemans G., Fréalle E., LGorton R., Guiver M., Hagen F. (2020). The Fungal PCR Initiative’s evaluation of in-house and commercial Pneumocystis jirovecii qPCR assays: Toward a standard for a diagnostics assay. Med. Mycol..

[13] Clancy C.J., Nguyen M.H. (2018). T2 magnetic resonance for the diagnosis of bloodstream infections: Charting a path forward. J. Antimicrob. Chemother..

[14] Clancy C.J., Pappas P.G., Vazquez J., Judson M.A., Kontoyiannis D.P., Thompson GR 3rd Garey K.W., Reboli A., Greenberg R.N., Apewokin S., Lyon GM 3rd Ostrosky-Zeichner L. (2018). Detecting Infections Rapidly and Easily for Candidemia Trial, Part 2 (DIRECT2): A Prospective, Multicenter Study of the T2Candida Panel. Clin. Infect. Dis..

[15] Muñoz P., Vena A., Machado M., Gioia F., Martínez-Jiménez M.C., Gómez E., Origüen J., Orellana M.Á., López-Medrano F., Fernández-Ruiz M. (2018). T2Candida MR as a predictor of outcome in patients with suspected invasive candidiasis starting empirical antifungal treatment: A prospective pilot study. J. Antimicrob. Chemother..

[16] Mylonakis E., Zacharioudakis I.M., Clancy C.J., Nguyen M.H., Pappas P.G. (2018). Efficacy of T2 Magnetic Resonance Assay in Monitoring Candidemia after Initiation of Antifungal Therapy: The Serial Therapeutic and Antifungal Monitoring Protocol (STAMP) Trial. J. Clin. Microbiol..

[17] Fuchs S., Lass-Flörl C., Posch W. (2019). Diagnostic Performance of a Novel Multiplex PCR Assay for Candidemia among ICU Patients. J. Fungi.

[18] Chien J.Y., Liu C.J., Chuang P.C., Lee T.F., Huang Y.T., Liao C.H., Hung C.C., Sheng W.H., Yu C.J., Hsueh P.R. (2017). Evaluation of the automated Becton Dickinson MAX real-time PCR platform for detection of Pneumocystis jirovecii. Future Microbiol..

[19] Schell W.A., Benton J.L., Smith P.B., Poore M., Rouse J.L., Boles D.J., Johnson M.D., Alexander B.D., Pamula V.K., Eckhardt A.E. (2012). Evaluation of a digital microfluidic real-time PCR platform to detect DNA of Candida albicans in blood. Eur. J. Clin. Microbiol. Infect. Dis..

[20] Sexton D.J., Bentz M.L., Welsh R.M., Litvintseva A.P. (2018). Evaluation of a new T2 Magnetic Resonance assay for rapid detection of emergent fungal pathogen Candida auris on clinical skin swab samples. Mycoses.

[21] Chong G.M., Vonk A.G., Meis J.F., Dingemans G.J., Houbraken J., Hagen F., Gaajetaan G.R., van Tegelen D.W., Simons G.F., Rijnders B.J. (2017). Interspecies discrimination of A. fumigatus and siblings A. lentulus and A. felis of the Aspergillus section Fumigati using the AsperGenius assay. Diagn. Microbiol. Infect Dis..

[22] White P.L., Posso R.B., Barnes R.A. (2015). Analytical and Clinical Evaluation of the PathoNostics AsperGenius Assay for Detection of Invasive Aspergillosis and Resistance to Azole Antifungal Drugs during Testing of Serum Samples. J. Clin. Microbiol..

[23] Wang Q., Kontoyiannis D.P., Li R., Chen W., Bu D., Liu W. (2019). A Novel Broad Allele-Specific TaqMan Real-Time PCR Method to Detect Triazole-Resistant Strains of Aspergillus fumigatus, even with a Very Low Percentage of Triazole-Resistant Cells Mixed with Triazole-Susceptible Cells. J. Clin. Microbiol..

[24] Chong G.L., van de Sande W.W., Dingemans G.J., Gaajetaan G.R., Vonk A.G., Hayette M.P., van Tegelen D.W., Simons G.F., Rijnders B.J. (2015). Validation of a new Aspergillus real-time PCR assay for direct detection of Aspergillus and azole resistance of Aspergillus fumigatus on bronchoalveolar lavage fluid. J. Clin. Microbiol..

[25] White P.L., Posso R.B., Barnes R.A. (2017). Analytical and Clinical Evaluation of the PathoNostics AsperGenius Assay for Detection of Invasive Aspergillosis and Resistance to Azole Antifungal Drugs directly from plasma Samples. J. Clin. Microbiol..

[26] Montesinos I., Delforge M.L., Ajjaham F., Brancart F., Hites M., Jacobs F., Denis O. (2017). Evaluation of a new commercial real-time PCR assay for diagnosis of Pneumocystis jirovecii pneumonia and identification of dihydropteroate synthase (DHPS) mutations. Diagn. Microbiol. Infect Dis..

[27] Yu L.S., Rodriguez-Manzano J., Malpartida-Cardenas K., Sewell T., Bader O., Armstrong-James D., Fisher M.C., Georgiou P. (2019). Rapid and Sensitive Detection of Azole-Resistant Aspergillus fumigatus by Tandem Repeat Loop-Mediated Isothermal Amplification. J. Mol. Diagn..

[28] Brown L., Rautemaa-Richardson R., Novak-Frazer L., Hill S., Hassan D., Richardson M.D., Gangneux J.-P., Lortholary O., Cornely O.A., Pagano L. Identification of Cyp51A-mediated triazole resistance in Aspergillus fumigatus using pyrosequencing: An audit of UK National Aspergillosis Centre cases. Proceedings of the 9th Trends in Medical Mycology.

[29] Van Der Torre M.H., Novak-Frazer L., Richardson R.-R. (2020). Detecting Azole-Antifungal Resistance in Aspergillus fumigatus by Pyrosequencing. J. Fungi.

[30] Arai T., Majima H., Watanabe A., Kamei K. (2020). A Simple Method to Detect Point Mutations in Aspergillus fumigatus cyp51A Gene Using a Surveyor Nuclease Assay. Antimicrob. Agents Chemother..

[31] Gutiérrez-Aguirre I., Rački N., Dreo T. (2015). Ravnikar Droplet digital PCR for absolute quantification of pathogens. Methods Mol. Biol..

[32] Poh T.Y., Ali N.A.B.M., Chan L.L.Y., Tiew P.Y., Chotirmall S.H. (2020). Evaluation of Droplet Digital Polymerase Chain Reaction (ddPCR) for the Absolute Quantification of Aspergillus species in the Human Airway. Int. J. Mol. Sci..

[33] Alanio A., Sturny-Leclere A., Benabou M., Guigue N., Bretagne S. (2016). Variation in copy number of the 28S rDNA of Aspergillus fumigatus measured by droplet digital PCR and analog quantitative real-time PCR. J. Microbiol. Methods.

[34] Kong H.H., Morris A. (2017). The emerging importance and challenges of the human mycobiome. Virulence.

[35] Krause R., Moissl-Eichinger C., Halwachs B., Gorkiewicz G., Berg G., Valentin T., Prattes J., Högenauer C., Zollner-Schwetz I. (2017). Mycobiome in the Lower Respiratory Tract—A Clinical Perspective. Front. Microbiol..

[36] Zoll J., Snelders E., Verweij P.E., Melchers W.J.G. (2016). Next-Generation Sequencing in the Mycology Lab. Curr. Fungal Infect. Rep..

[37] Bittinger K., Charlson E.S., Loy E., Shirley D.J., Haas A.R., Laughlin A., Yi Y., Wu G.D., Lewis J.D., Frank I. (2014). Improved characterization of medically relevant fungi in the human respiratory tract using next-generation sequencing. Genome Biol..

[38] Mukherjee P.K., Chandra J., Retuerto M., Sikaroodi M., Brown R.E., Jurevic R., Salata R.A., Lederman M.M., Gillevet P.M. (2014). Ghannoum Oral mycobiome analysis of HIV-infected patients: Identification of Pichia as an antagonist of opportunistic fungi. PLoS Pathog..

[39] Delhaes L., Monchy S., Fréalle E., Hubans C., Salleron J., Leroy S., Prevotat A., Wallet F., Wallaert B., Dei-Cas E. (2012). The Airway Microbiota in Cystic Fibrosis: A Complex Fungal and Bacterial Community—Implications for Therapeutic Management. PLoS ONE.

[40] Vu D., Groenewald M., De Vries M., Gehrmann T., Stielow B., Eberhardt U., Al-Hatmi A., Groenewald J.Z., Cardinali G., Houbraken J. (2018). Large-scale generation and analysis of filamentous fungal DNA barcodes boosts coverage for kingdom Fungi and reveals thresholds for fungal species and higher taxon delimitation. Stud. Mycol..

[41] Nilsson R.H., Anslan S., Bahram M., Wurzbacher C., Baldrian P., Tedersoo L. (2019). Mycobiome diversity: High-throughput sequencing and identification of fungi. Nat. Rev. Genet..

[42] Abdolrasouli A., Rhodes J., Beale M.A., Hagen F., Rogers T.R., Chowdhary A., Meis J.F., Armstrong-James D., Fisher M.C. (2015). Genomic Context of Azole Resistance Mutations in Aspergillus fumigatus Determined Using Whole-Genome Sequencing. mBio.

[43] Garnaud C., Botterel F., Sertour N., Bougnoux M.E., Dannaoui E., Larrat S., Hennequin C., Guinea J., Cornet M., Maubon D. (2015). Next-generation sequencing offers new insights into the resistance of Candida spp. to echinocandins and azoles. J. Antimicrob Chemother..

[44] Fisher C.E., Hohl T.M., Fan W., Storer B.E., Levine D.M., Zhao L.P., Martin P.J., Warren E.H., Boeckh M., Hansen J.A. (2017). Validation of single nucleotide polymorphisms in invasive aspergillosis following hematopoietic cell transplantation. Blood.

[45] Campos C.F., Van De Veerdonk F.L., Gonçalves S.M., Cunha C., Netea M.G., Carvalho A. (2018). Host Genetic Signatures of Susceptibility to Fungal Disease. Curr. Top. Microbiol. Immunol..

[46] White P.L., Parr C., Barnes R.A. (2017). Predicting Invasive Aspergillosis in Hematology Patients by Combining Clinical and Genetic Risk Factors with Early Diagnostic Biomarkers. J. Clin. Microbiol..

[47] D’Ordine R.L., Garcia K.A., Roy J., Zhang Y., Markley B., Finkelman M.A. (2020). Performance characteristics of Fungitell STAT^TM^, a rapid (1→3)-β-D-glucan single patient sample in vitro diagnostic assay. Med. Mycol..

[48] Mercier T., Guldentops E., Patteet S., Beuselinck K., Lagrou K., Maertens J. (2019). Beta-D-Glucan for Diagnosing Pneumocystis Pneumonia: A Direct Comparison between the Wako β-Glucan Assay and the Fungitell Assay. J. Clin. Microbiol..

[49] Friedrich R., Rappold E., Bogdan C., Held J. (2018). Comparative Analysis of the Wako beta-Glucan Test and the Fungitell Assay for Diagnosis of Candidemia and Pneumocystis jirovecii Pneumonia. J. Clin. Microbiol..

[50] De Carolis E., Marchionni F., Torelli R., Angela M.G., Pagano L., Murri R., De Pascale G., De Angelis G., Sanguinetti M., Posteraro B. (2020). Comparative performance evaluation of Wako beta-glucan test and Fungitell assay for the diagnosis of invasive fungal diseases. PLoS ONE.

[51] Leeflang M., Debets-Ossenkopp Y.J., Visser C.E., Scholten R.J.P.M., Hooft L., Bijlmer H.A., Reitsma J.B., Bossuyt P.M.M., Vandenbroucke-Grauls C.M. (2008). Galactomannan detection for invasive aspergillosis in immunocompromized patients. Cochrane Database Syst. Rev..

[52] Gorton R.L., White P.L., Bagkeris E., Cotterall D., Desai R., McHugh T., Kibbler C.C. (2015). Improved Standardization of the Bio-Rad Platelia Aspergillus Galactomannan Antigen Sandwich Enzyme Immunoassay Using the DS2 (Dynex) Enzyme-Linked Immunosorbent Assay (ELISA) Processing System. J. Clin. Microbiol..

[53] White P.L., Price J.S., Posso R., Vale L., Backx M. (2020). An Evaluation of the Performance of the IMMY Aspergillus Galactomannan Enzyme-Linked Immunosorbent Assay When Testing Serum to Aid in the Diagnosis of Invasive Aspergillosis. J. Clin. Microbiol..

[54] Dichtl K., Seybold U., Ormanns S., Horns H., Wagener J. (2019). Evaluation of a Novel *Aspergillus* Antigen Enzyme-Linked Immunosorbent Assay. J. Clin. Microbiol..

[55] Huang H.R., Fan L.C., Rajbanshi B., Xu J.F. (2015). Evaluation of a new cryptococcal antigen lateral flow immunoassay in serum, cerebrospinal fluid and urine for the diagnosis of cryptococcosis: A meta-analysis and systematic review. PLoS ONE.

[56] Thornton C.R. (2008). Development of an immunochromatographic lateral-flow device for rapid serodiagnosis of invasive aspergillosis. Clin. Vaccine Immunol..

[57] Pan Z., Fu M., Zhang J., Zhou H., Fu Y., Zhou J. (2015). Diagnostic accuracy of a novel lateral-flow device in invasive aspergillosis: A meta-analysis. J. Med Microbiol..

[58] Mercier T., Dunbar A., de Kort E., Schauwvlieghe A., Reynders M., Guldentops E., Blijlevens N.M.A., Vonk A.G., Rijnders B., Verweij P.E. (2020). Lateral flow assays for diagnosing invasive pulmonary aspergillosis in adult hematology patients: A comparative multicenter study. Med. Mycol..

[59] Lass-Flörl C., Lo Cascio G., Nucci M., Camargo Dos Santos M., Colombo A.L., Vossen M., Willinger B. (2019). Respiratory specimens and the diagnostic accuracy of Aspergillus lateral flow assays (LFA-IMMY™): Real-life data from a multicentre study. Clin. Microbiol. Infect..

[60] White P.L., Price J.S., Posso R., Cutlan-Vaughan M., Vale L., Backx M. (2020). An evaluation of the performance of the IMMY sona Aspergillus Galactomannan Lateral flow assay when testing serum to aid in the diagnosis of invasive aspergillosis. J. Clin. Microbiol..

[61] Marr K.A., Datta K., Mehta S., Ostrander D.B., Rock M., Francis J., Feldmesser M. (2018). Urine Antigen Detection as an Aid to Diagnose Invasive Aspergillosis. Clin. Infect. Dis..

[62] Johnson G., Shannon M., Thornton C., Agrawal S., Lass-Floerl C., Mutschlechner W. Proximity ligation assay for the early detection of invasive aspergillosis. Proceedings of the 25th European Congress of Clinical Microbiology and Infectious Diseases.

[63] Mery A., Sendid B., François N., Cornu M., Poissy J., Guerardel Y., Poulain D. (2016). Application of Mass Spectrometry Technology to Early Diagnosis of Invasive Fungal Infections. J. Clin. Microbiol..

[64] Sato K., Oinuma K.-I., Niki M., Yamagoe S., Miyazaki Y., Asai K., Yamada K., Hirata K., Kaneko Y., Kakeya H. (2017). Identification of a Novel Rhizopus-specific Antigen by Screening with a Signal Sequence Trap and Evaluation as a Possible Diagnostic Marker of Mucormycosis. Med. Mycol..

[65] Shibata W., Niki M., Sato K., Fujimoto H., Yamada K., Watanabe T., Miyazaki Y., Asai K., Obata Y., Tachibana T. (2020). Detection of Rhizopus-specific antigen in human and murine serum and bronchoalveolar lavage. Med. Mycol..

[66] Jambunathan K., Watson D.S., Najvar L.K., Wiederhold N.P., Kirkpatrick W.R., Patterson T.F., Askew D.S., Kodukula K., Galande A.K. (2013). Prolyl endopeptidase activity in bronchoalveolar lavage fluid: A novel diagnostic biomarker in a guinea pig model of invasive pulmonary aspergillosis. Med. Mycol..

[67] Abad-Diaz-De-Cerio A., Fernandez-Molina J.V., Ramirez-Garcia A., Sendino J., Hernando F.L., Pemán J., Garaizar J., Rementeria A. (2013). The aspHS gene as a new target for detecting Aspergillus fumigatus during infections by quantitative real-time PCR. Med. Mycol..

[68] Morton C.O., White P.L., Barnes R.A., Klingspor L., Cuenca-Estrella M., Lagrou K., Bretagne S., Melchers W., Mengoli C., Caliendo A.M. (2017). Determining the analytical specificity of PCR-based assays for the diagnosis of IA: What is Aspergillus?. Med. Mycol..

[69] Bächer P., Steinbach A., Kniemeyer O., Hamprecht A., Assenmacher M., Vehreschild M.J.G.T., Vehreschild J.J., Brakhage A.A., Cornely O.A., Scheffold A. (2015). Fungus-Specific CD4+T Cells for Rapid Identification of Invasive Pulmonary Mold Infection. Am. J. Respir. Crit. Care Med..

[70] Potenza L., Vallerini D., Barozzi P., Riva G., Gilioli A., Forghieri F., Candoni A., Cesaro S., Quadrelli C., Maertens J. (2016). Mucorales-Specific T Cells in Patients with Hematologic Malignancies. PLoS ONE.

[71] Lauruschkat C.D., Wurster S., Page L., Lazariotou M., Dragan M., Weis P., Ullmann A.J., Einsele H., Löffler J. (2018). Susceptibility of A. fumigatus-specific T-cell assays to pre-analytic blood storage and PBMC cryopreservation greatly depends on readout platform and analytes. Mycoses.

[72] Syhre M., Scotter J.M., Chambers S.T. (2008). Investigation into the production of 2-Pentylfuran by Aspergillus fumigatus and other respiratory pathogens in vitro and human breath samples. Med. Mycol..

[73] De Heer K., van der Schee M.P., Zwinderman K., van den Berk I.A., Visser C.E., van Oers R., Sterk P.J. (2013). Electronic nose technology for detection of invasive pulmonary aspergillosis in prolonged chemotherapy-induced neutropenia: A proof-of-principle study. J. Clin. Microbiol..

[74] Bhimji A., Bhaskaran A., Singer L.G., Kumar D., Humar A., Pavan R., Lipton J., Kuruvilla J., Schuh A., Yee K. (2018). Aspergillus galactomannan detection in exhaled breath condensate compared to bronchoalveolar lavage fluid for the diagnosis of invasive aspergillosis in immunocompromised patients. Clin. Microbiol. Infect..

[75] Florio W., Tavanti A., Ghelardi E., Lupetti A. (2018). MALDI-TOF MS Applications to the Detection of Antifungal Resistance: State of the Art and Future Perspectives. Front. Microbiol..

[76] Vatanshenassan M., Boekhout T., Lass-Flörl C., Lackner M., Schubert S., Kostrzewa M., Sparbier K. (2018). Proof of Concept for MBT ASTRA, a Rapid Matrix-Assisted Laser Desorption Ionization-Time of Flight Mass Spectrometry (MALDI-TOF MS)-Based Method to Detect Caspofungin Resistance in Candida albicans and Candida glabrata. J. Clin. Microbiol..

[77] Leelahavanichkul A., Worasilchai N., Wannalerdsakun S., Jutivorakool K., Somparn P., Issara-Amphorn J., Tachaboon S., Srisawat N., Finkelman M.A., Chindamporn A. (2016). Gastrointestinal Leakage Detected by Serum (1→3)-β-D-Glucan in Mouse Models and a Pilot Study in Patients with Sepsis. Shock.

[78] Gianella S., Letendre S.L., Iudicello J., Franklin D., Gaufin T., Zhang Y., Porrachia M., Vargas-Meneses M., Ellis R.J., Finkelman M. (2019). Plasma (1 → 3)-β-d-glucan and suPAR levels correlate with neurocognitive performance in people living with HIV on antiretroviral therapy: A CHARTER analysis. J. Neurovirol..

[79] White P.L., Posso R., Parr C., Price J.S., Finkelman M., Barnes R.A. (2020). The presence of (1-3)-β-D-Glucan as prognostic marker in patients post major abdominal surgery. Clin. Infect. Dis..

[80] Agnelli C., Bouza E., Martínez-Jiménez M.D.C., Navarro R., Valerio M., Machado M., Guinea J., Sánchez-Carrillo C., Alonso R., Muñoz P. (2019). Clinical Relevance and Prognostic Value of Persistently Negative (1,3)-β-D-Glucan in Adults with Candidemia: A 5-year Experience in a Tertiary Hospital. Clin. Infect. Dis..

[81] Rautemaa-Richardson R., Rautemaa V., Al-Wathiqi F., Moore C.B., Craig L., Felton T.W., Muldoon E.G. (2018). Impact of a diagnostics-driven antifungal stewardship programme in a UK tertiary referral teaching hospital. J. Antimicrob. Chemother..

[82] Donnelly J.P., Chen S.C., Kauffman C.A., Steinbach W.J., Baddley J.W., Verweij P.E., Clancy C.J., Wingard J.R., Lockhart S.R., Groll A.H. (2020). Revision and Update of the Consensus Definitions of Invasive Fungal Disease From the European Organization for Research and Treatment of Cancer and the Mycoses Study Group Education and Research Consortium. Clin. Infect. Dis..

